# Is It Possible to Optimize the Elaboration and Preservation of a Vancomycin Catheter Lock Solution?

**DOI:** 10.3390/antibiotics14060605

**Published:** 2025-06-14

**Authors:** Marta Díaz-Navarro, David Samitier, Félix García-Moreno, María Sanjurjo, Patricia Muñoz, Beatriz Torroba-Sanz, María Guembe

**Affiliations:** 1 Clinical Microbiology and Infectious Diseases, Gregorio Marañón Hospital, 28007 Madrid, Spain; marta.diaz@iisgm.com (M.D.-N.); pmunoz@hggm.es (P.M.); 2 Instituto de Investigación Sanitaria Gregorio Marañón, 28007 Madrid, Spain; david.samitier@salud.madrid.org (D.S.); felixjesus.garcia@salud.madrid.org (F.G.-M.); maria.sanjurjo@salud.madrid.org (M.S.); beatriz.torroba@salud.madrid.org (B.T.-S.); 3 Pharmacy Department, Gregorio Marañón Hospital, 28007 Madrid, Spain; 4 Faculty of Medicine, Universidad Complutense de Madrid, 28040 Madrid, Spain

**Keywords:** vancomycin, catheter lock, preservation, concentration, biofilm

## Abstract

**Background/Objectives**: Vancomycin (V) is widely used for catheter lock therapy. However, its ad hoc preparation in pharmacy departments involves discarding most of an intravenous vial and contributes to high workload. We aimed to assess the V concentration and minimum inhibitory biofilm concentration (MIBC) of a frozen V lock solution. **Methods**: Two V-2 mg/mL solutions were tested: (1) V + heparin 100 IU/mL and (2) V + citrate 2%. Solutions were frozen at −20 °C, followed by 48 h refrigeration, and analyses were performed at baseline and after 2, 4, 8, and 12 weeks (experiment 1). In addition, after the 12-week freezing period, solution 1 was also preserved for 1 and 2 weeks at both 4 °C and room temperature (experiment 2). V concentration was assessed by HPLC-DAD at 205 nm and validated with forced degradation tests. A <10% variation indicated significant change. MBIC was determined by XTT staining of 24 h biofilms exposed to decreasing concentrations of each solution. Microorganisms tested included methicillin-susceptible and -resistant *Staphylococcus aureus* (MSSA, MRSA), *Staphylococcus epidermidis* ATCC35984 (SE), and a highly biofilm-forming clinical *S. epidermidis* strain (SEclin). MIBC was defined as ≥50% reduction in metabolic activity. **Results**: In experiment 1, while V concentration remained stable over time, MIBC values varied, notably increasing from 8 weeks for all strains. Moreover, in experiment 2, significant reductions in both V concentration and MIBC were detected in the 2-week period. **Conclusions**: V lock solution appears to be able to be 12-weeks frozen followed by up to 1 week at refrigeration or room temperature. This facilitates the optimization of vial preparation in hospital pharmacy laboratories.

## 1. Introduction

Guidelines for the management of catheter-related bloodstream infection (C-RBSI) recommend the combination of systemic antibiotics and lock therapy when catheter removal is not possible and the patient is stable and uncomplicated [1]. This approach helps prevent catheter-related infections and maintain patency, but it carries risks such as drug resistance, toxicity, and the potential for systemic side effects. Vancomycin (V) is the most commonly used antibiotic for catheter locks that are infected with *Staphylococcus* spp., which are the most common microorganisms causing C-RBSI, followed by Gram-negative bacilli and *Candida* spp. [2]. The ad hoc elaboration of a catheter lock solution’s vials requires individual preparation by the pharmacy department, which not only involves discarding a vial for IV administration of which only a few mL are needed, but such on-demand preparation also creates a high workload. Based on previous studies of our group with V and dalbavancin solutions, it was therefore possible to demonstrate that these solutions can be prepared using the entire vial and divided into single-dose vials to be kept frozen and individually thawed if necessary [3,4,5,6]. However, only the anti-biofilm capacity was assessed by quantification of the reduction in metabolic activity and the V lock solution concentration used was 5 mg/mL. Therefore, it is necessary to determine in a 2 mg/mL V lock solution not only the concentration of V in the frozen solution but also whether there are changes in the minimum inhibitory biofilm concentration (MIBC).

## 2. Results

### 2.1. Experiment 1

**Figure 1:**
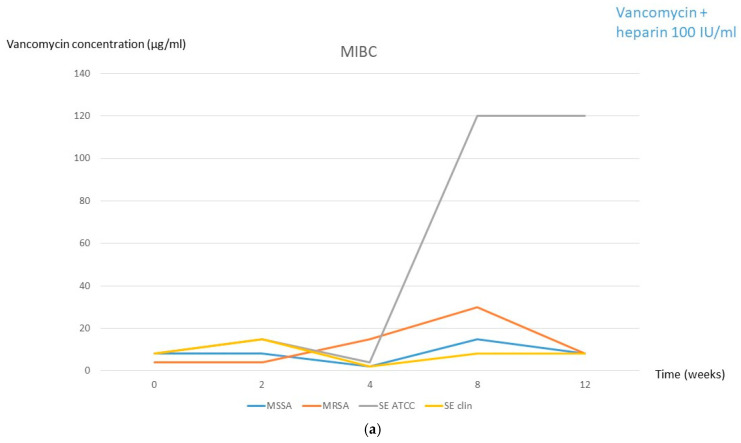

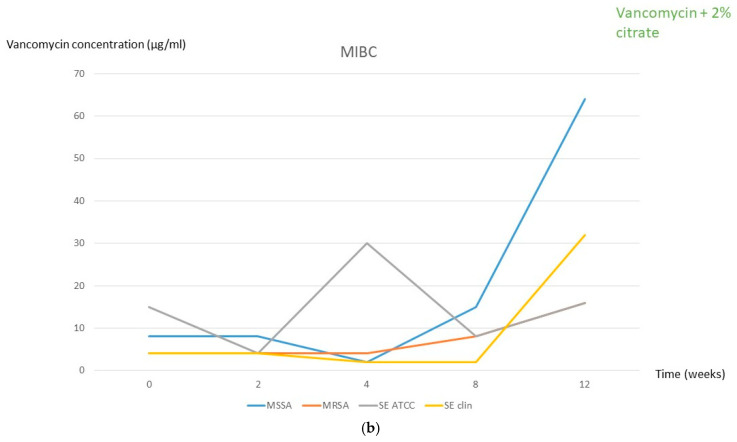
MIBC at each frozen period time followed by 2 days in the fridge for both solutions. (**a**) Solution 1 (2 mg/mL vancomycin + 100 IU/mL heparin). (**b**) Solution 2 (2 mg/mL vancomycin + 2% citrate). **MSSA**, methicillin-susceptible *Staphylococcus aureus*; **MRSA**, methicillin-resistant *Staphylococcus aureus*; **SE**, *Staphylococcus epidermidis*; **clin**, clinical; **MIBC**, minimum inhibitory biofilm concentration.

During freezing for solution 1, no significant oscillation in the MIBC was observed, except for the ATCC strain of S. epidermidis, which increased to 120 µg/mL from week 4 (Figure 1a, Table 1 and Table 2). In solution 2, the MIBC of the S. epidermidis ATCC strain was also slightly increased from week 4 (30 µg/mL) and the MIBC of the three remaining strains also increased from week 8 (Figure 1b, Table 1 and Table 2).

**Figure 2:**
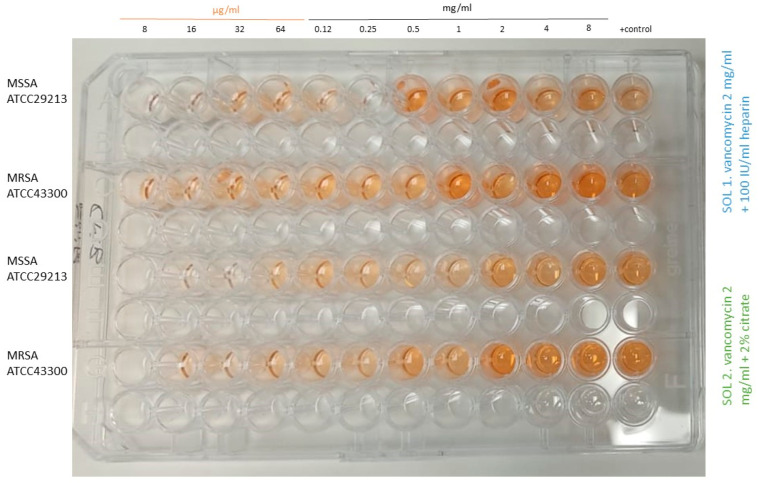
Paradoxical effect in MSSA and MRSA at time 0 for both tested solutions from 8 µg/mL to 8 mg/mL. **MSSA**, methicillin-susceptible *Staphylococcus aureus*; **MRSA**, methicillin-resistant *Staphylococcus aureus*; **sol**, solution.

One interesting finding in this experiment was that we observed a paradoxical effect (Eagle effect), especially in *S. aureus*, when planktonic cells were exposed to increasing concentrations of vancomycin from 8 µg/mL to 8 mg/mL (combined either with heparin or citrate). Figure 2 shows this effect in MSSA and MRSA of both solutions at time 0, as an example. It can be observed that at low concentrations, the metabolic activity was almost absent, whereas from 0.5 to 8 mg/mL it proportionally increased, reaching the same colour (or even higher) than the positive control.

### 2.2. Experiment 2

**Figure 3:**
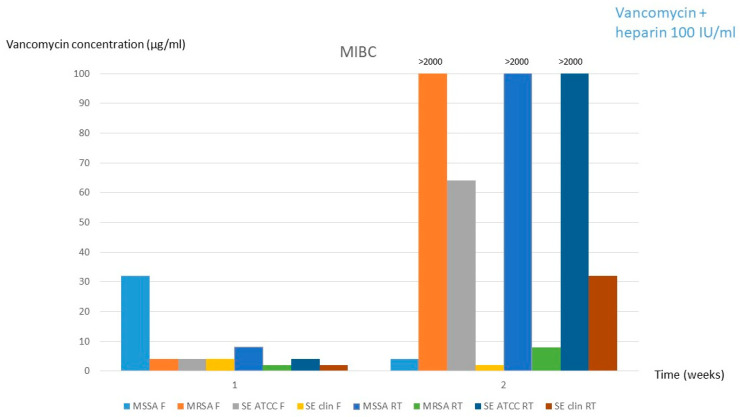
MIBC for solution 1 (2 mg/mL vancomycin + 100 IU/mL heparin) after the 12-weeks frozen period followed by 1 and 2 weeks at fridge or room temperature. **MSSA**, methicillin-susceptible *Staphylococcus aureus*; **MRSA**, methicillin-resistant *Staphylococcus aureus*; **SE**, *Staphylococcus epidermidis*; **clin**, clinical; **MIBC**, minimum inhibitory biofilm concentration; **F**, fridge; **RT**, room temperature.

Based on the similar results obtained for both solutions in experiment 1, experiment 2 was only performed with solution 1. As it can be observed in Table 1 and Table 2, significant changes in vancomycin concentration (<limit of significance of 10%) occurred only at the 2-week period both in refrigeration and room temperature (1.38 mg/mL and 1.48 mg/mL, respectively). Moreover, the MIBC was also significantly increased to >2000 mg/mL in room temperature conservation for MSSA and *S. epidermidis* ATCC and in fridge conservation for MRSA (Table 1 and Table 2, Figure 3).

## 3. Discussion

As we previously demonstrated that the efficacy of V was not affected in 5 mg/mL lock solution after a 6-month freezing period [6,7], we have now tested the V concentration and MIBC of a 2 mg/mL V lock solution both with 100 IU/mL of heparin and 2% citrate in different scenarios of conservation and preservation, demonstrating that they can be frozen up 12 weeks followed by a 48 h refrigeration. Moreover, in the vancomycin–heparin solution, it can be extended to a 1-week preservation at 4 °C or room temperature.

We observed that, although the concentration of vancomycin remained unchanged throughout the 12-week freezing + 48 h refrigeration periods in both solutions, the MIBC fluctuated, with a significant increase from week 8 onwards, mainly with solution 2, in both Staphylococci spp. However, none of the MIBC reached the concentration value of the lock solution prepared for clinical administration (2 mg/mL). In contrast, when solution 1 was frozen for 12 weeks followed by 2 weeks of either refrigeration or room temperature, both the solution 1 concentration and the MIBC suffered significant changes. Thus, the best strategy for optimizing a V lock solution is to preserve it under freezing for 12 weeks in pharmacy departments followed by 1 week of either refrigeration or room temperature maintenance in nursing departments.

It is important to highlight the paradoxical effect that we observed during our experiments. This Eagle effect was first described by Eagle in 1948 with penicillin, which was then also observed by Straton et al. in 1987 [8,9] and Stille et al. with *Enterococcus* in 1973 [10]. Recently, this effect was also reported with echinocandins and *Candida* [11]. Regarding vancomycin, there is only one study, of Jarrad et al., in which they observed the Eagle effect with *C. difficile* strains in an in vitro study [12]. Therefore, ours is the first study assessing the vancomycin Eagle effect in Staphylococcal strains, although what remains unclear is its clinical impact. Ericson et al. found no correlation between high doses of ampicillin and worse clinical outcomes in children with bacteremic [13]. In contrast, Griffiths et al. demonstrated clinical failure with penicillin [14]. Price et al. observed a paradoxical relationship between vancomycin MIC and outcomes of *S. aureus* bacteremia patients [15]. However, in candidemia, Rueda et al. demonstrated that the presence of the paradoxical effect was not associated with a patient’s response to antifungal treatment [16]. Our concern is whether the paradoxical effect of vancomycin can have any negative impact in patient outcomes when used as a catheter lock solution. Guidelines recommend using a vancomycin concentration at least 1,000 times higher than the MIC (5 mg/mL) [1] and clinical studies assessing the clinical impact and efficacy of vancomycin lock therapy (VLT) are somewhat discrepant, mainly because of different criteria being used to define the success of VLT [17,18]. Our group previously described that the success of catheter retention in 76 staphylococcal C-RBSI episodes using VLT was moderate, reaching slightly more than 70% when the catheter was kept in place until the end of use [3]. Worse results have been reported in a recent randomized study (ETHALOCK) of Lesens et al. in which success rates were 46.7% and 58.1% in patients treated with 5 mg/mL VLT and 40% ethanol lock therapy, respectively [19]. In line with the low success rates of VLT, recently, Permuy et al. described three cases of failure in children who were finally successfully treated with daptomycin [20]. This was also observed in a recent study of Blanco Di-Mateo et al. where daptomycin locks achieved the highest eradication rate of coagulase-negative Staphylococci (CoNS) from 21 haemodialysis catheters, with vancomycin being the one showing the worst results [21]. Moreover, in another recent study in 100 cancer patients with totally implantable venous access devices infected with CoNS, the success rate was slightly low after 3 months of VLT (44.0%) [22]. These poor success rates of VLT can be explained in part because of its well-known low anti-biofilm activity, as better results with other lipoglycopeptides have been shown [23,24,25,26,27]. In particular, in the quantitative in vitro model of Kropec et al., the activity of vancomycin and teicoplanin in two concentrations (4× MIBC and 1 µg/mL) against *S. aureus* and *S. epidermidis* strains colonizing the internal surface of polyurethane and silicone catheters was studied. They showed that teicoplanin achieved a significantly greater reduction (*p* < 0.05) in the counts of both strains compared to vancomycin [28]. However, Liang et al. used a lower concentration of heparin-based vancomycin lock solution (25 µg/mL) for 137 very low birth weight infants with peripherally inserted central venous catheters to prevent C-RBSI, showing a statistical difference in the C-RBSI rates between infants treated with vancomycin locks (n = 68) and single heparin locks (n = 69, control) (4.4% vs. 21.7%, *p* = 0.004) [29]. This high difference in the C-RBSI rates between groups may be explained because they used lower concentrations of vancomycin, which may have avoided the possible negative effect of using high concentrations, based on our described Eagle effect occurring from 30 µg/mL onwards.

Finally, the main advantages of this preservation approach in hospital practice include improved efficiency in compounding processes, allowing the number of syringes per batch and the frequency of preparation to be adjusted according to actual demand. Additionally, it will help to reduce the operational burden on pharmacy staff by extending the interval between preparation cycles from every 3–4 days to every 24–30 days. This strategy also helps avoid waste from expired lock solutions, which was previously common due to their limited shelf life. In contrast, the main limitations of this approach include the “intermediate risk” classification of the preparation, which requires adherence to strict aseptic conditions and quality controls. Additionally, although our study assessed physicochemical and therapeutic stability, it did not evaluate microbiological stability. According to current guidelines, this limits the maximum beyond-use date in frozen storage to 45 days. Extending this period would require microbiological testing to ensure safety. Moreover, freezing and thawing processes must be standardized to avoid variability in the final product [30].

## 4. Materials and Methods

Two V-2 mg/mL solutions were tested: (1) V + heparin 100 IU/mL (heparina sódica Sala 5000 UI/5 mL, Reig Jofre, Sant Joan Despi, Spain) and (2) V + citrate 2% (Citra-Lock TM 4% 5mL, B. Braun, Melsungen, Germany). We used 2 mg/mL instead of 5 mg/mL because 2 mg/mL is the standard in our institution based on the physical compatibility with heparin. Two experiments were performed: Experiment 1: V solutions were frozen at −20 °C, followed by 48 h refrigeration at 4 °C, and analyses were performed at baseline and after 2, 4, 8, and 12 weeks. In addition, based on the similar results obtained for both solutions in experiment 1 and considering that heparin is the anticoagulant used mostly in clinical practice and in our institution, experiment 2 was only performed with solution 1, which consisted of a 12-week freezing period followed by 1 and 2 weeks of both 4 °C refrigeration and room temperature periods.

### 4.1. Concentration Test

The pH of the V lock solutions were determined in triplicate. The concentration of V was tested by HPLC-DAD (Agilent 1 Infinity II, Agilent Technologics, Santa Clara, CA, USA). Method validation was performed by forced degradation tests under acidic, alkaline, and oxidizing conditions. The linearity of the calibration line was confirmed at concentrations of 125–250 µg/mL (R2 = 0.989 y = 227.41x + 700). The retention time of V was 4.1 min. The stationary phase was a C18 column and the mobile phase was an acetonitrile–aqueous potassium phosphate mixture (0.05 M, pH = 3.1) at a rate of 0.5 µL/min and a concentration gradient of T° 40 °C. Samples were diluted 1:10, 1 µL was injected in triplicate, and detection was set at 205 nm. Alterations <10% (1.80 mg/mL) were considered to indicate a significant change in sample composition.

### 4.2. Minimum Inhibitory Biofilm Concentration (MIBC)

MIBC was assessed by the measurement of metabolic activity by tetrazolium salt (XTT) staining (Merck, Spain) in 24 h biofilm in well plates by spectrophotometry (EZ READ 400, Biochrom, Spain) (treated with decreasing concentrations from 8 µg/mL to 8000 µg/mL of sol 1 and sol 2 vs. positive control) of each of the following microorganisms: methicillin-susceptible *Staphylococcus aureus* (MSSA ATCC29213), methicillin-resistant *S. aureus* (MRSA ATCC43300), *Staphylococcus epidermidis* (ATCC35984), and a biofilm-forming clinical strain of *S. epidermidis* obtained from the blood of a patient with C-RBSI that we had previously used in other experimental studies because it showed high biomass production. MIBC was considered when the reduction in metabolic activity was ≥50% when contrasting the XTT absorbance of treated vs. positive control wells. We considered significant alteration of MIBC change to have occurred when it reached the vancomycin lock solution concentration of 2 mg/mL. No other statistical analysis could be performed comparing MIBC at different times because only one single value of absorbance was obtained and no mean/median could be compared.

## 5. Conclusions

Despite that vancomycin lock solution can be frozen up 12 weeks, preservation at 4 °C or room temperature can only be carried out up to 1 week when used in combination with heparin. Even so, this strategy can help to optimize the elaboration and preservation of vancomycin lock solutions in pharmacy and nursing departments. This approach may serve to reduce risks due to variability in preparation and regulatory considerations.

## Figures and Tables

**Table 1:** Vancomycin concentration and MBEC during the study periods.

Time	Solution *	[ ] mg/mL	MIBC µg/mL			
			MSSA	MRSA	SE ATCC	SE Clinical
**0**	1	2.00	8	4	8	8
	2	2.00	8	4	15	4
**2w −80 °C + 48 h F**	1	1.91	8	4	15	15
	2	2.05	8	4	4	4
**4w −80 °C + 48 h F**	1	1.86	2	15	4	2
	2	1.92	2	4	30	2
**8w −80 °C + 48 h F**	1	1.91	15	30	120	8
	2	1.95	15	8	8	2
**12w −80 °C + 48 h F**	1	1.94	8	8	120	8
	2	2.02	64	16	16	32
**12w −80 °C+ 1w F**	1	1.86	32	4	4	4
**12w −80 °C + 1w RT**	1	1.86	8	2	4	2
**12w −80 °C + 2w F**	1	**1.38**	4	>2000 **	64	2
**12w −80 °C + 2w RT**	1	**1.48**	>2000 **	8	>2000 **	32

**MSSA**, methicillin-susceptible *Staphylococcus aureus*; **MRSA**, methicillin-resistant *Staphylococcus aureus*; **SE**, *Staphylococcus epidermidis*; **w**, weeks; **[ ]**, concentration; **MIBC**, minimum inhibitory biofilm concentration; **F**, fridge; **RT**, room temperature. * Solution 1: vancomycin + heparin 100 UI/mL. Solution 2: vancomycin + 2% citrate. ** Overcame the vancomycin lock solution concentration of 2 mg/mL = 2000 µg/mL. Values in bold represent that the concentration exceeds the established limit of significance of a 10% reduction in vancomycin lock solution concentration (<1.80 mg/mL).

**Table 2:** MIBC fold dilution changes compared to the basal experiment (t = 0) according to the four tested strains treated with frozen vancomycin solutions during the study periods.

Strain	MIBC Fold Dilutions Changes											
	Weeks at −80 °C											
	2 w + 48 h F		4 w + 48 h F		8 w + 48 h F		12 w + 48 h F		12 w + 1 w F	12 w + 2 w F	12 w + 1 w RT	12 w + 2 w RT
	sol 1	sol 2	sol 1	sol 2	sol 1	sol 2	sol 1	sol 2	sol 1	sol 1	sol 1	sol 1
MSSA	=	=	2 ↓	2 ↓	**1 ↑**	**1 ↑**	=	**3 ↑**	**2 ↑**	1 ↓	=	↑ >8 *
MRSA	=	=	**2 ↑**	=	**3 ↑**	**1 ↑**	**1 ↑**	**2 ↑**	=	↑ >8 *	1 ↓	**1 ↑**
SE ATCC	**1 ↑**	2 ↓	1 ↓	**1 ↑**	**4 ↑**	1 ↓	**4 ↑**	=	1 ↓	**↑** **3**	1 ↓	↑ >8 *
SE clinical	**1 ↑**	=	2 ↓	1 ↓	=	1 ↓	**3 ↑**	**3 ↑**	1 ↓	2 ↓	2 ↓	**2 ↑**

**MSSA**, methicillin-susceptible *Staphylococcus aureus*; **MRSA**, methicillin-resistant *Staphylococcus aureus*; **SE**, *Staphylococcus epidermidis*; **w,** weeks; **MIBC**, minimum inhibitory biofilm concentration; **F**, fridge; **RT**, room temperature; **sol**, solution. Values in bold represent those dilutions that increased compared to the baseline value at time 0. * Overcame the vancomycin lock solution concentration of 2 mg/mL = 2000 µg/mL.

## Data Availability

The original contributions presented in this study are included in the article. Further inquiries can be directed to the corresponding author.

## Abbreviations

The following abbreviations are used in this manuscript:

V	Vancomycin
VLT	Vancomycin lock therapy
C-RBSI	Catheter-related bloodstream infection
MIBC	Minimum inhibitory biofilm concentration
IQR	Interquartile range
XTT	Tetrazolium salt
MSSA	Methicillin-susceptible *Staphylococcus aureus*
MRSA	Methicillin-resistant *Staphylococcus aureus*
SE	*Staphylococcus epidermidis*
Ok.SEclin	*Staphylococcus epidermidis* clinical strain
CoNS	Coagulase-negative Staphylococci
